# Assessing the link between witnessing inter-parental violence and the perpetration of intimate partner violence in Bangladesh

**DOI:** 10.1186/s12889-017-4067-4

**Published:** 2017-02-10

**Authors:** Md. Jahirul Islam, Mosiur Rahman, Lisa Broidy, Syed Emdadul Haque, Yu Mon Saw, Nguyen Huu Chau Duc, Md. Nurruzzaman Haque, Md. Mostafizur Rahman, Md. Rafiqul Islam, Md. Golam Mostofa

**Affiliations:** 1 School of Criminology and Criminal Justice, Griffith University, Mt Gravatt Campus , 176 Messines Ridge Road, Brisbane, 4122 QLD Australia; 2 Ministry of Planning, Bangladesh Planning Commission, Sher-e-Bangla Nagar, Dhaka, 1207 Bangladesh; 3 Department of Population Science and Human Resource Development, University of Rajshahi, Rajshahi, 6205 Bangladesh; 4 Department of Sociology, 1 University of New Mexico, Albuquerque, NM 87131 USA; 5 United Nations University-International Institute for Global Health (UNU-IIGH), Kuala Lumpur, Malaysia; 6 Department of Healthcare Administration, Graduate School of Medicine, Nagoya University , Nagoya, Japan; 7 Department of Paediatrics, Hue University of Medicine and Pharmacy, Hue University , Hue, Vietnam

**Keywords:** Witnessing inter-parental violence, IPV perpetration, Justification of wife-beating, Men, Intergenerational transmission of violence, Bangladesh

## Abstract

**Background:**

We aimed to examine the influence of witnessing father-to-mother violence on: 1) perpetration of intimate partner violence (IPV); and 2) endorsement of attitudes justifying wife beating in Bangladesh.

**Methods:**

This paper used data from the 2007 Bangladesh Demographic Health Survey. The analyses were based on the responses of 3374 ever-married men. Exposure to IPV was determined by men’s self-reports of witnessing inter-parental violence in childhood. We used adjusted binary logistic regression models to assess the influence of exposure on husbands’ perpetration of IPV and their endorsement of attitudes justifying wife beating.

**Results:**

Nearly 60% of men reported violent behaviour towards an intimate partner and 35.7% endorsed attitudes justifying spousal abuse. Men who witnessed father-to-mother violence had higher odds of reporting any physical or sexual IPV (adjusted OR [AOR] = 3.26; 95% CI = 2.61, 4.06). Men who had witnessed father-to-mother violence were also 1.34 times (95% CI = 1.08, 1.65) more likely endorse attitudes justifying spousal abuse.

**Conclusions:**

Committing violence against an intimate partner is an all too frequent practice among men in Bangladesh. The study indicated that men who had witnessed father-to-mother violence were more likley to perpetrate IPV, suggesting an intergenerational transmission of violence. This transmission of violence may operate through the learning and modelling of attitudes favourable to spousal abuse. In support of this, witnnessing inter-parental violence was also associated with the endorsement of attitudes justifying spousal abuse. Our findings indicate the continued importance of efforts to identify and assist boys who have witnessed domestic violence and suggest such efforts should aim to change not just behaviours but also attitudes that facilitate such violence.

## Background

Intimate partner violence (IPV), which consists of a range of physically and sexually coercive acts perpetrated in the context of an intimate relationship [1], has emerged as a significant public health concern around the world. While both males and females are victims of IPV, the paternalistic culture of Bangladesh reinforces traditional gender paradigms and puts women at particularly high risk for IPV victimization. In fact, IPV affects 69% of Bangladeshi women during their lifetimes [2]. There has been an increased awareness of the wide range of mental, physical, sexual, and reproductive health consequences of IPV [3–8]. Given the alarmingly high prevalence of IPV and the serious physical, psychological, and interpersonal consequences experienced by victims of such abuse, it is vital to identify risk factors for perpetrating IPV. Addressing these risk factors can reduce not just conseuqneces of IPV but its actual prevalence.

There is evidence that broad risk factors associated with all manner of poor outcomes are also associated with the perpetration of IPV. These include environmental deficits, low socio-economic status, substance use, mental illness, and poor parenting [9–12]. However, there are other more specific risk factors that might serve as better flags for IPV specific prevention initiatives. Particularly for males, witnessing inter-parental violence has long been considered one of the central psychosocial risk factors that lead to perpetration of IPV in martial relationships, and is also one of the key factors implicated in the inter-generational transmission of partner violence.

The association between witnessing IPV in childhood and later perpetration has been found in multiple studies in diverse settings [13–17], with the overwhelming majority of data derived from high-income countries—primarily the United States. There is a dearth of evidence from developing countries. However, most of the studies in developing countries have looked at the intergenerational transmission of IPV *victimization* among women, with little research attention to how witnessing inter-parental violence affects subsequent IPV *perpetration* among males. For example, a study carried out in Nigeria found that women who ever witnessed inter-parental violence were reported to have tolerant attitudes toward IPV against women, and were more likely to be abused by their spouse than those who had not witnesses parental IPV [18]. Another study from Pakistan concluded that women who reported that their mothers were beaten by their fathers were at increased risk for IPV victimization, and were more likely than those without exposure to parental IPV to agree that beating is justified if a wife argues with her husband [19]. These findings suggest that part of the intergenerational transmission of violence may be linked to the transmission of attitudes that support such violence and increase vulnerability to IPV victimization in developing countries. Less clear is whether witnessing parental IPV similarly shapes men’s attitudes in these settings, increasing their likelihood of IPV perpetration.

Until recently women have been the primary focus of secondary prevention efforts, with the aim of supporting women at risk for IPV victimization. However, recent policy efforts recognize that IPV rates are unlikely to change substantially if interventions do not also address the attitudes and behaviours that increase IPV perpetration among men. There is a paucity of research regarding intergenerational transmission of violence in Bangladesh, and evidence regarding men’s attitudes towards the justification of spousal abuse is limited. As is the case in other developing countries, the only study on this topic in Bangladesh focuses on women’s experiences as victims [20]. This study concluded that women who witnessed parental IPV were more likely to experience IPV later in their marital life. To the best of our knowledge no studies in Bangladesh have explicitly explored how witnessing parental violence affects the behaviour and attitudes of Bangladeshi men. Based on these considerations, we examine the importance of inter-parental violence witnessed by men in their childhood to assess its potential links to various forms of IPV perpetration. Using the nationally representative survey of ever-married Bangladeshi men, we examine whether witnessing father-to-mother violence is linked to: 1) men’s perpetration of violence against an intimate partner; and 2) men’s endorsement of attitudes justifying spousal abuse.

## Methods

### Data sources

The analyses were based on the representative, cross sectional 2007 Bangladesh Demographic Health Survey (BDHS) data. We used the 2007 BDHS data since after that BDHS did not include violence module in the later survey of BDHS 2011 [21]. The BDHS sample was drawn from Bangladeshi adults residing in private dwellings, and it measures indicators of population, health, and nutrition. The BDHS uses extensive interviewer training, standardized measurement tools and techniques, an identical core questionnaire, and instrument pretesting to ensure standardization and comparability across diverse sites and time. The survey employed a stratified, multi-stage cluster sample of 361 primary sampling units (134 in urban areas and 227 in rural areas). The primary sampling units were derived from a sampling frame created for the 2001 Bangladeshi census. A primary sampling unit, which consists of about 100 households, on average, is equivalent to a mauza in rural areas and to a mohallah in urban areas. On average, 30 households were selected from each PSU, using an equal probability systematic sampling technique. In this way, 10,819 households were selected for the sample

**Fig. 1:**
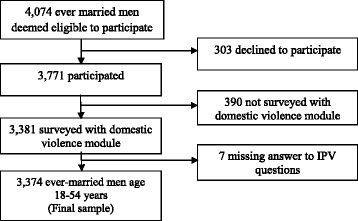
Selection of sample. From the original 4074 eligible men, we obtained a final sample of 3374, ever married men for this study, 2007 Bangladesh Demographic Health Survey

The 2007 BDHS used five questionnaires: household, women, men, community, and a facilities questionnaire. Their contents were based on the MEASURE DHS model questionnaires. The questionnaires were developed in English and then translated and printed in the official language, Bangla. The men’s questionnaire was used to collect information from men aged 18–54 years who had ever married. Of 4074 eligible men identified, 3771 participated (92.6% response rate). Questions on domestic violence were administered to only one eligible respondent per household [21]. Of the 3381 men eligible to respond to the domestic violence module, only seven had to be excluded owing to lack of privacy or because they declined participation. We use this sample of ever-married men aged 18–54 years (*n* = 3374; Fig. 1) for our analyses.

### Outcomes

Perpetration of IPV and husband’s endorsement of attitudes justifying spousal abuse are the outcomes of interest in this study. After consulting with experts, the 2007 BDHS used a standardized approach to the measurement of IPV, specifically the shortened and modified version of the Conflict Tactics Scale (CTS-2) [22]. The 2007 BDHS stated that to ensure validity and reliability of the data collected on IPV, fieldworkers underwent careful training in different aspects of interview techniques and the questionnaires were pre-tested in pilot studies [21]. Moreover, to make valid cross-national comparisons, the questionnaire used to measure IPV in the BDHS include the same criteria and methods used in other cultural contexts.

Following the CTS-2 protocol, IPV was assessed through eight items pertaining to lifetime perpetration of violence against women. Focusing on lifetime estimates of IPV provides information about the burden of violence within a population rather than a snapshot of the recent burden of violence. For our purposes, we are interested in the broad links between early exposure to parental IPV, attitudes that justify IPV, and any perpetration, rather than the situational and contextual factors that might explain specific IPV events. For this reason, lifetime estimates are a more appropriate outcome than recent IPV perpetration. Respondents were coded as perpetrators of physical IPV if they had reported ever: pushing, shaking or throwing an object; (2) slapping; (3) pulling hair or twisting an arm; (4) punching or hitting with a fist or something harmful; (5) kicking or dragging; (6) choking or burning; or (7) threatening or attacking their wife with a knife or gun. The Cronbach’s α for this measure was 0.83. Perpetration of sexual IPV was indicated by a man’s positive response to an item asking whether he had physically forced his wife to have sexual intercourse even when she did not want to. Men’s responses were used to create a 4-level categorical variable reflecting 3 categories of IPV: physical IPV only, sexual IPV only, and both physical and sexual IPV. The fourth category was a referent category indicating no IPV perpetration of either form. In addition, we created a binary variable measuring whether a man reported perpetration of any form of IPV (physical, sexual, or both).

Husband’s attitudes towards the justification of spousal abuse was measured based on the men’s answers to five questions regarding the conditions under which beating or raping ones’ wife would be justifiable. These include: 1) if she goes out without informing her husband; 2) if she argues with her husband; 3) if she neglects the children; 4) if she refuses to have sex with her husband; and 5) if she neglects the elder. For each of these questions, response options regarding whether beating or raping would be justified in this situation were yes (1) or no (0). We created a bivariate measure that separates those who did not feel beating or raping a spouse is justifiable under any condition (0) from those who endorsed beating or raping a spouse under one or more of the listed conditions (1).

### Exposure

Witnessing of father-to-mother violence is the primary exposure of interest in this study. We use a single item measure that reflects whether the respondent’s father beat his mother ever (yes = 2; no = 1).

### Control variables

We included several socio-demographic variables theoretically and empirically linked to perpetration of IPV [11, 23, 24] and acceptance of spousal abuse [25]. We classified participants by their current age into three broad groups: young adults (1 = 18–33 years), middle aged (2 = 34–43years) and older (3 = 44–54 years). The men’s educational level was defined in terms of the formal education system of Bangladesh: no education (1 = 0 year), primary (2 = 1–5 years) or secondary or higher (3 = 6 years or more). Place of residence was categorized as rural (1) versus urban (2). To assess women’s decision-making autonomy in the respondent’s household, we used a continuous measure that ranges from 0 to 5 and reflects the number of types of family decisions the respondent’s wife makes alone or jointly (with the husband), including whether to obtain health care for herself, to make large purchases, to make household purchases, to visit her relatives, and decision on use of wife’s earning. This measure reflects the husband’s endorsement of his wife’s autonomy. We created a variable for respondent’s occupation and classified men according to whether they were involved in manual labour (1 = agricultural workers, fisherman, home-based manufacturing, poultry, cattle raising, domestic servant, rickshaw polar, factory workers) or were working in a non-manual profession (2 = doctors, lawyers, engineers, teachers or large business men). Total numbers of household members were classified in tertiles (1 = 2–4; 2 = 5–6; 3 = 7 or more). Religion was categorized as Muslim (2) or non-Muslim (1). A relative index of household wealth was calculated based on interviewer-observed assets, including ownership of consumer items and the characteristics of the dwelling place; individual household wealth scores were assigned to the poorest (1), middle (2), or richest (3) tertile. Whether respondents’ earnings meet the basic family needs was broken into three groups: insufficient (1), moderately sufficient (2) or sufficient (3). Whether the respondent ever used drugs or alcohol was categorized as no (1) versus yes (2). Marital duration was broken into four categories (1 = 0–4 years; 2 = 5–9 years; 3 = 10–14 years; 4 = 15 years or more).

### Statistical analyses

We calculated descriptive statistics for our sample’s socio-demographic characteristics as well as IPV exposure and men’s attitudes towards the justification of spousal abuse. We used *χ*2 analyses to assess socio-demographic differences in IPV perpetration reported by men against their wife. In all the analyses, the level of significance was set at *p* < 0.05 (two tailed). We then created three multivariate logistic regression models. Two binary logistic regressions were used for our binary outcomes: any physical and/or sexual IPV and endorsement of attitudes justifying spousal abuse. We used multinomial logistic regression to analyse our nominal outcome variable: separate effects of physical IPV only, sexual IPV only and both physical and sexual IPV as compared to no IPV. We ran an additional multivariate model to see whether men who *both* witnessed inter-parental violence and endorsed attitudes justifying spousal abuse were more likely to commit violence against women than were those who report one or the other. This model includes a four-category variable that divides the sample of men into the following groups: men who have both witnessed inter-parental violence and endorse attitudes justifying spousal abuse; men who have witnessed inter-parental violence only; men who endorse attitudes justifying spousal abuse only; and those who report neither witnessing violence nor endorsing attitudes that justify violence.

We checked all models for multicollinearity by examining variance inflation factors (VIFs), but find no evidence that this is a problem (VIF’s <2.0 for all models). The odds ratios (ORs) in the binary models and relative risk ratio (RRRs) in the multinomial models were estimated to assess the strength of the associations, with 95% confidence intervals (CIs) defined as the benchmark for significance testing. All the covariates were entered simultaneously into the multiple regression models. We used imputation, based on a regression model, to estimate missing values from known values to account for missing data. Age, education of respondent, and place of residence were included as covariates in the imputation. However imputation of missing data did not affect our results since only <1% missing covariates were imputed. All analyses were weighted to account for the complex sampling design of the BDHS and analyses were all run using Stata (Stata, version 11.0; Stata-Corp, College Station, Texas). This study was considered to be exempt from a full review as it was based on an anonymous public use of a secondary data set with no identifiable information regarding the survey participants.

## Results

### Descriptive statistics

Table 1 presents sample descriptive and evaluates variation in IPV perpetration as a function of socio-demographic characteristics as well as men’s endorsement of attitudes justifying spousal abuse. Focusing first on sample descriptive (Table 1; Column 1), approximately 34.0% participants were 44–54 years old, 30.5% had no education, 90.3% were Muslims, and 77.2% lived in rural areas at the time of the survey. There was a fairly even income distribution across respondents and approximately 62.0% of the respondents reported that their earnings were moderately sufficeint to meet the basic family needs. The majority of respondents (73.2%) worked non-manual jobs. The modal household size was 2–4 members (37.9%). Very few participants reported using drugs or alcohol (3.1%), a common risk factor for IPV in Western settings. Finally, the overwhelming majority of men (95%) reported that their wives had at least some decision-making autonomy in the household.

**Table 1:** Descriptive statistics, according to different forms of IPV against their wives committed by ever married men and their endorsement of attitudes justifying spousal abuse: 2007 Bangladesh Demographic Health Survey (*n* = 3374)

Characteristic	n (%)	Any IPV	Physical IPV only	Sexual IPV only	Both physical and sexual IPV	Endorsement of attitudes justifying spousal abuse
		% (95% CI)	% (95% CI)	% (95% CI)	% (95% CI)	% (95% CI)
Age, years						
18-33	1107 (35.3)	53.8 (50.1-57.6)	43.5 (39.8-47.3)	2.0 (1.2-3.4)	8.4 (6.0-11.6)	39.2 (35.0-43.7)
34-43	1136 (31.2)	63.5 (59.6-67.2)	54.0 (50.4-57.5)	1.9 (1.1-3.4)	7.6 (5.7-10.0)	34.4 (30.8-38.3)
44-54	1131 (33.5)	62.1 (58.7-65.5)	55.2 (51.4-59.0)	1.5 (0.8-2.9)	5.4 (3.9-7.6)	33.2 (30.0-36.9)
*p* Value		<0.001	<0.001	0.721	0.128	0.068
Education						
No education	998 (30.5)	67.8 (64.2-71.2)	58.0 (53.9-62.0)	1.1 (0.5-2.4)	8.7 (6.4-11.8)	41.2 (37.3-45.2)
Primary	1078 (32.8)	65.4 (62.0-68.7)	56.5 (52.8-60.2)	1.7 (0.8-3.4)	7.2 (5.5-9.3)	40.4 (36.7-44.3)
Secondary and higher	1298 (36.6)	47.6 (44.0-51.3)	39.4 (36.1-42.8)	2.5 (1.6-3.9)	5.8 (3.7-8.8)	26.9 (23.7-30.4)
*p* Value		<0.001	<0.001	0.193	0.219	<0.001
Area of residence						
Rural	2100 (77.2)	61.1 (58.6-63.7)	51.7 (49.0-54.4)	2.0 (1.3-3.0)	7.5 (5.8-9.5)	38.3 (35.5-41.1)
Urban	1274 (22.8)	54.5 (50.3-58.6)	47.3 (43.1-51.5)	1.3 (0.8-2.2)	6.0 (4.4-8.1)	27.0 (23.5-30.8)
*p* Value		0.008	0.080	0.234	0.255	<0.001
Religion						
Non-Muslims	340 (9.7)	51.2 (44.6-57.7)	43.7 (37.1-50.6)	2.6 (1.1-6.1)	4.8 (2.3-9.7)	38.4 (32.1-45.4)
Muslims	3034 (90.3)	60.5 (58.2-62.8)	51.5 (49.0-53.9)	1.7 (1.1-2.6)	7.4 (5.9-9.1)	35.4 (33.0-37.8)
*p* Value		0.007	0.037	0.383	0.256	0.374
Wealth index category						
Poor	1124 (36.1)	66.5 (62.8-70.0)	56.9 (53.2-60.6)	1.3 (0.7-2.4)	8.3 (6.4-10.7)	44.0 (40.2-47.9)
Middle	1125 (34.9)	61.0 (56.9-64.9)	52.2 (48.2-56.1)	1.9 (1.0-3.5)	6.9 (4.9-9.6)	38.6 (35.0-42.3)
Rich	1125 (29.0)	49.5 (45.4-53.6)	41.1 (36.9-45.5)	2.4 (1.4-4.2)	5.9 (3.3-10.5)	219 (18.4-25.7)
*p* Value		<0.001	<0.001	0.314	0478	<0.001
Respondent’s occupation^a^						
Non-manual	2382 (73.2)	62.8 (60.3-65.2)	53.6 (51.0-56.2)	1.8 (1.1-2.8)	7.4 (6.0-9.1)	39.6 (36.8-42.9)
Manual	992 (26.8)	51.0 (46.5-55.5)	42.8 (38.4-47.3)	1.9 (1.1-3.4)	6.3 (4.1-9.5)	25.1 (218–29.0)
*p* Value		<0.001	<0.001	0.821	0.433	<0.001
Earning provide family basic needs						
Sufficient	394 (11.2)	50.8 (44.5-57.0)	41.2 (35.7-47.0)	2.8 (1.3-5.6)	6.8 (4.3-10.5)	28.5 (23.7-33.9)
Moderately sufficient	2061 (61.6)	58.2 (55.4-60.9)	49.7 (47.1-52.4)	2.1 (1.4-3.2)	6.4 (4.8-8.3)	33.8 (31.0-36.6)
Insufficient	919 (27.2)	66.5 (62.5-70.2)	56.8 (52.4-61.1)	0.7 (0.2-1.7)	9.0 (6.7-12.0)	43.0 (38.4-47.8)
*p* Value		<0.001	<0.001	0.026	0.121	<0.001
Any living children						
No	345 (11.1)	35.2 (29.1-41.8)	26.3 (21.3-32.1)	2.9 (1.3-6.4)	5.9 (2.6-13.1)	33.6 (27.9-39.9)
Yes	3029 (88.9)	62.7 (60.3-65.0)	53.7 (51.3-56.2)	1.7 (1.1-2.4)	7.3 (5.9-8.9)	36.0 (33.6-38.4)
*p* Value		<0.001	<0.001	0.170	0.630	0.458
No. of household members (tertiles)						
2-4	1374 (37.9)	62.5 (59.3-65.8)	53.3 (50.1-56.5)	1.8 (1.1-2.8)	7.5 (5.9-9.6)	38.5 (35.0-42.0)
5-6	1185 (34.7)	61.0 (57.6-64.2)	52.4 (48.9-55.9)	1.8 (0.9-3.5)	6.7 (5.0-9.0)	34.0 (30.9-37.4)
≥ 7	815 (27.4)	53.8 (49.4-58.2)	44.9 (40.5-50.0)	1.8 (0.9-3.9)	7.1 (4.5-10.9)	34.0 (29.7-38.6)
*p* Value		0.003	0.004	0.996	0.851	0.120
Wives’ decision-making autonomy, no. of aspects^b^						
0	122 (4.9)	32.8 (23.5-43.7)	27.9 (19.4-38.4)	2.7 (0.4-.7)	2.2 (0.7-6.9)	38.8 (28.6-50.2)
1	174 (5.9)	46.8 (36.6-57.1)	36.1 (27.1-46.3)	2.9 (0.1-8.4)	7.7 (4.9-14.4)	33.9 (24.5-44.8)
2	155 (5.3)	45.0 (35.9-54.5)	35.4 (27.0-44.9)	4.0 (1.5-10.6)	5.6 (2.5-11.9)	31.7 (23.5-41.1)
3	195 (6.4)	56.4 (46.2-66.0)	48.5 (38.6-58.4)	1.5 (0.4-5.2)	6.5 (1.9-20.2)	31.7 (24.6-39.8)
4	2088 (5.9)	62.3 (59.6-65.0)	53.6 (50.7-56.4)	1.4 (0.8-2.2)	7.4 (5.7-9.5)	35.6 (33.0-38.3)
5	640 (19.0)	67.4 (62.7-71.9)	57.3 (52.7-61.7)	2.1 (1.1-4.0)	8.1 (5.9-10.8)	38.1 (33.5-43.0)
*p* Value		<0.001	<0.001	0.442	0.576	0.688
Ever use drug or alcohol^c^						
No	3262 (96.9)	59.2 (56.9-61.4)	50.6 (48.3-52.9)	1.7 (1.2-2.5)	6.9 (5.5-8.5)	35.2 (32.9-37.6)
Yes	112 (3.1)	73.9 (64.5-81.6)	53.3 (42.3-64.0)	4.9 (1.7-13.1)	15.7 (9.9-24.0)	50.6 (40.5-60.7)
*p* Value		0.004	0.635	0.046	0.001	0.003
Marital duration, years						
0-4	614 (19.3)	41.9 (37.0-47.0)	33.3 (28.9-38.0)	2.9 (1.5-5.5)	5.8 (3.2-10.2)	39.1 (33.7-44.7)
5-9	649 (19.0)	60.4 (55.7-65.0)	50.4 (45.7-55.1)	1.8 (0.9-3.5)	8.2 (5.3-12.4)	38.3 (33.6-43.3)
10-14	565 (15.5)	65.7 (61.0-70.3)	54.4 (49.4-59.2)	2.1 (1.0-4.5)	9.2 (6.6-12.8)	33.8 (29.2-38.8)
≥ 15	1546 (46.1)	64.7 (61.6-67.6)	56.9 (53.6-60.1)	1.3 (0.7-2.4)	6.5 (5.0-8.5)	33.9 (30.8-37.1)
*p* Value		<0.001	<0.001	0.223	0.350	0.175
Witnessed father-to-mother IPV						
No	2505 (73.5)	53.2 (50.6-55.8)	46.5 (43.9-49.1)	1.7 (1.1-2.7)	5.0 (3.9-6.4)	32.4 (30.0-35.1)
Yes	869 (26.5)	77.5 (74.0-80.8)	62.4 (58.0-66.6)	2.1 (1.1-3.8)	13.1 (9.9-17.0)	44.8 (40.4-49.3)
*p* Value		<0.001	<0.001	0.669	<0.001	<0.001
Prevalence		59.6	50.7	1.8	7.1	35.7

*Note: p* Values refer to differences between groups. Numbers are unweighted; percentages are weighted

IPV intimate partner violence

^a^ Manual labor includes agricultural workers, fisherman, home-based manufacturing, poultry, cattle raising, domestic servant, rickshaw puller, factory workers and non-manual workers include professional workers (doctors, lawyers, engineers, teachers), large business

^b^ Aspects of family decisions a woman participated alone or jointly in making

^c^ Ghanja, charas, phensdile, pethedine, heroin, morphin, injectable drugs, other drugs

With respect to our central measures of interest, a substantial percentage of men (59.6%) reported ever commiting physical or sexual violence against their wife; 50.7% indicated that they had committed only physical IPV, 1.8% indicated that they had committed only sexual IPV and 7.1% indicated that they had commited both types of IPV. Approximately 27.0% of men reported that they had witnessed father-to-mother violence. Nearly 36.0% endorsed attitudes justifying spousal abuse.

Table 1 also shows the socio-demographic differentials for different forms of IPV commited by men against their wives and their endorsement of attitudes justifying spousal abuse. Significantly, and consistent with expectations, the prevalence of any physical or sexual IPV, physical IPV only, both physical and sexual IPV, and endorsement of attitudes justifying spousal abuse was higher among those men who had witnessed father-to-mother violence. We also find that all of the control variables are associated with total IPV and with physical IPV, but not with sexual IPV or both physical and sexual IPV. We suspect this is becauses the prevalence of sexual IPV is relatively low, masking any variation across groups. In any case, these difference reinforce the importance of controlling for these demographic characteristics when evaluating whether witnessing parental IPV and endorsing attitudes that justify spousal abuse impact on IPV perpetration.

#### Multivaraite analysis

Table 2 shows the results of multivariate analyses assessing the influence of witnessing parental IPV and of endorsing attitudes that justify spousal abuse on different forms of IPV committed by ever married men. In the adjusted binary logistic regression model, men who reported witnessing father-to-mother violence were significantly more likely to also report any physical or sexual IPV (adjusted OR [AOR] = 3.26; 95% CI = 2.61, 4.06). Men who had witnessed father-to-mother violence were also 1.34 times (95% CI = 1.08, 1.65) more likely to have endorse justifying spousal abuse. The multinomial logistic regression analysis shows that for men who had witnessd father-to-mother violence, compared to those who did not, the relative risk for physical IPV only, sexual IPV only, and both physical and sexual IPV relative to no IPV would be expected to increase by a factor of 3.17 (95% CI = 2.53, 3.96), 2.40 (95% CI = 1.04,5.58), and 5.97 (95% cI = 4.05, 8.81) respectively, given the other variables in the model are held constant.

**Table 2:** Multivariate analysis for different forms of IPV committed by ever married men and their endorsement of attitudes justifying spousal abuse with witnessing father-to mother IPV: 2007 Bangladesh Demographic Health Survey (*n* = 3374)

Measure	Any IPV^1^	Types of IPV^2^			Endorsement of attitudes justifying spousal abuse^3^
		RRR (95% CI)^§^			
	AOR (95% CI)^§^	Only physical IPV	Only sexual IPV	Both physical and sexual IPV	AOR (95% CI)^¥^
Witnessed father-to-mother IPV					
No	1.00	1.00	1.00	1.00	1.00
Yes	3.26 (2.61-4.06)^a^	3.17 (2.53-3.96)^a^	2.40 (1.04-5.58)^c^	5.97 (4.05-8.81)^a^	1.34 (1.08-1.65)^b^
Interaction of witnessing inter-parental violence AND endorsing attitudes justifying spousal abuse					
Both witnnessing inter-parental violence and endorsing attitudes justifying spousal abuse	1.00				
Witnnessing inter-parental violence only	0.41 (0.25-0.65)^a^				
Endorsing attiudes justifying spousal abuse only	0.29 (0.19-0.45)^a^				
None	0.13 (0.08-0.19)^a^				

Note: ^1,2,3^ Models were adjusted for age, education, place of residence, religion, wealth index category, earning provide family basic needs, any living children, number of family members, decision making autonomy, ever use of drug or alcohol, and marital duration

^§^Adjusted for endorsement of attitudes justifying spousal abuse^3^

^¥^Adjusted for any IPV

*AOR* adjusted odds ratio, *RRR* relative risk ratio, *CI* confidence interval, *IPV* intimate partner violence

^a^ *p* < 0.001, ^b^ *p* < 0.01, ^c^ *p* < 0.05

Table 2 also shows the association of the interaction between witnessing parental IPV and attitudes toward the justification of spousal abuse with IPV. In the adjusted binary logistic regression model, men who only reported witnessing father-to-mother violence (AOR = 0.41 95% CI = 0.25, 0.65) or who only endorsed attitudes justifying spousal abuse (AOR = 0.29; 95% CI = 0.19, 0.45) were significantly less likely to report any physical or sexual IPV compared to the men who reported *both* witnessing father-to-mother violence and endorsing attitudes justifying spousal abuse.

## Discussion

The results of this large and nationally representative survey in Bangladesh showed that nearly 60% of ever-married men committed physical or sexual violence against their wives and 35.7% endorsed attitudes justifying spousal abuse. Having witnessed inter-parental violence increased the likelihood that these men endorsed attitudes justifying spousal abuse. Further, multivariate models confirmed that men who witnessed of father-to-mother violence were more likely to report IPV perpetration. Also important, those who both witnessed inter-parental IPV and endorsed attitudes justifying spousal abuse were exhibited the highest likelihood of perpetrating IPV.

The high lifetime prevalence rate of IPV revealed in this study confirms that perpetration of IPV is a shockingly frequent practice among men in this impoverished South Asian nation, and that it potentially affects the health of a large majority of Bangladeshi women. Furthermore, consistent with prior IPV research conducted in developing nations, including Bangladesh, IPV perpetration was most prevalent among the most disadvantaged strata of Bangladeshi society.

To the best of our knowledge, this is the first study in Bangladesh that uses data from a nationally representative sample to examine the link between witnessing inter-parental violence, attitudes towards spousal abuse, and IPV perpetration from a male perspective. The findings that witnessing inter-parental violence increases the likelihood of physical or sexual IPV agrees with some of the previous studies, which indicated that boys who witness inter-parental conflict are more likely to approve of violence, to believe that violence improves their reputation, and to justify their own violent behaviour, compared with boys who have not witnessed such violence [13, 17, 26].

There are many mechanisms through which witnessing inter-parental violence by men could be related to increased likelihood for committing violence against women. Evidence shows that men exposed to inter-parental violence have high negative emotional reactivity, behavioural dysregulation, externalizing problems, and lower IQs [27, 28]. Many of these factors relate to perpetration of IPV [29], because of perpetrators are more likely to attribute hostile intent, to view violence as acceptable, and to have lower verbal and social skills and poorer marital communication than non-perpetrators [29]. Men who observed inter-parental violence remember the contexts and sequences of the incidents of their parents’ violence [16]. These cognitions, as memories, remain etched (and transmitted) inter-generationally and set expectations for the outcomes of a man’s violent behaviour against his wife [16]. In other words, boys (who saw their fathers beat their mothers) may assert that it is acceptable for a man to beat his female partner, because it conforms to the behaviours and attitudes displayed by their fathers. Moreover, evidence shows that, a history of witnessing inter-parental violence is highly correlated with drug or alcohol abuse, poverty, and poor educational achievement [30, 31], which are strong predictors of violence in later life. We performed additional analyses (results available on request) that support this hypothesis; in addition to an increased likelihood of IPV perpetration, men who had witnessed inter-parental violence were found to have a higher prevalence of drug or alcohol use, have little education, be manual labourers, and be poor.

It is also important to highlight our findings regarding the interactive role of experiences and attitudes on behaviour. Not only do we find that witnessing inter-parental violence makes men more likely to endorse attitudes justifying spousal abuse, and that both independently increase the odds of IPV, we also find an interactive effect. Men who report both witnessing IPV and endorsing attitudes that justify spousal abuse exhibit the highest likelihood of perpetrating IPV when compared to those who experience one or the other (or neither). This finding comports with expectation from social learning theory. Social learning theory conceptualizes violence against women and attitudes towards the spousal abuse as learned behaviours that result from socialization or modelling. In one US study, individuals who directly experienced abuse as a child or who witnessed abuse inflicted on another female in the house (e.g., their mother) were significantly more likely to endorse stereotypical gender-role attitudes and to believe that violence is appropriate for conflict resolution and is acceptable in intimate interpersonal settings [16]. Other research from the US suggests a noteworthy relationship between early childhood experiences with family violence and individuals’ attitudes as adults and acceptance of spousal abuse [32, 33]. Our findings reinforce this literature and suggest these links are similarly prominent in Bangladesh.

A key strength of this study is the use of a nationally representative sample of Bangladeshi men to examine the link between witnessing inter-parental violence with attitudes towards spousal abuse and IPV perpetration from a male perspective. Despite the use of a large, nationally representative sample of ever married men in Bangladesh, the results of the current study should be considered in light of some limitations. First, due to the cross-sectional nature of this analysis, temporal order cannot be confirmed. Longitudinal and qualitative research is needed to clarify the causal and temporal relationships between IPV and witnessing inter-parental violence. That said, it is unlikely that witnessing parental IPV would be subsequent to a respondent’s own marriage and IPV perpetration. More problematic is the temporal relationship between attitudes justifying spousal abuse and perpetration of spousal abuse. Though it makes theoretical sense that attitudes would precede behaviour, it is possible that men create or endorse justifications for abuse after engaging in the behaviour. While counter to learning theory, it would be consistent with other theoretical frameworks, specifically, Sykes and Matza’s techniques of neutralization. Again, panel data would be necessary to properly sort causal order in this case. Second, although psychological violence is an important facet of IPV, information on this form of violence was not available from the BDHS. Third, the questionnaire assessed witnessing of inter-parental violence through a single question of the mother’s experience of physical violence (“Did your father ever beat your mother?”). There is a possibility of under-estimation of the effect since in some cases men may be unaware of violence between their parents. Moreover, we cannot discern when or how often this violence occurred and its frequency, severity or recency may affect its influence on men’s subsequent attitudes and behaviours. Finally, because data were self-reported, men may under-report their use of IPV. However the prevalence of IPV obtained in our study is similar with women’s reports of lifetime IPV (48.0%) in 2007 BDHS [21]. Moreover, the personal interview method used in this study is widely employed for this type of IPV research. In addition, to ascertain physical and sexual IPV, BDHS followed established best-practices by using multiple, behaviourally specific questions.

## Conclusion

Violence against women at the hands of their husbands is all too frequent in Bangladesh. Using a nationally representative sample of ever married men from Bangladesh, we find that men who had witnessed of father-to-mother violence were particularly likely to report perpetrating IPV. Witnessing inter-parental violence was also associated with positive attitudes towards the justification of spousal abuse. Findings also indicate that men who both witnessed inter-parental violence and endorsed attitudes justifying spousal abuse were among the most likely to commit violence against women. This suggests that witnessing abuse affects subsequent IPV not just through behavioural modeling but also through the transmission of cultural messages that support spousal abuse. Our finding of an association of witnessing inter-parental violence with perpetration of violence against women, after adjusting for a wide range of potential confounders, indicates that efforts to identify and assist individuals who have witnessed such violence should continue to receive support. However, our finding that 65.9% of perpetrators reported not having witnessed inter-parental conflict signifies the need to understand other risk and protective factors across the lifespan, particularly those that may be modified to support nonviolent behaviour. Future longitudinal studies are needed to investigate the causal link between witnessing inter-parental violence and the perpetration of IPV. Our research, though, suggests that attitudes that support such behaviour are among the key modifiable mechanisms that policies and programs should address.

## Abbreviations

AOR: Adjusted odds ratio
BDHS: Bangladesh Demographic and Health Survey
CI: Confidence interval
CTS: Conflict tactics scale
IPV: Intimate partner violence
RRR: Relative risk ratio
VIF: Variance inflation factors

## References

[1] World Health Origination. World Report on Violence. Geneva: WHO; 1997. www.who.int/whr/1998/en/whr98_en.pdf. Accessed 30 Aug 2012.

[2] Heise L, Garcia-Moreno C, Krug EG, Dahlberg LL, Mercy JA, Zwi AB, Lozano R (2002). Violence by intimate partners. World Report on Violence and Health.

[3] Rahman M1, Nakamura K, Seino K, Kizuki M (2012). Intimate partner violence and use of reproductive health services among married women: evidence from a national Bangladeshi sample. BMC Public Health.

[4] Rahman M1, Nakamura K, Seino K, Kizuki M (2013). Intimate partner violence and chronic undernutrition among married Bangladeshi women of reproductive age: are the poor uniquely disadvantaged?. Eur J Clin Nutr.

[5] Rahman M1, Nakamura K, Seino K, Kizuki M (2014). Intimate partner violence and symptoms of sexually transmitted infections: are the women from low socio-economic strata in Bangladesh at increased risk. Int J Behav Med.

[6] Rahman M1, Nakamura K, Seino K, Kizuki M (2013). Are survivors of intimate partner violence more likely to experience complications around delivery? Evidence from a national Bangladeshi sample. Eur J Contracept Reprod Health Care.

[7] Ellsberg M, Jansen HA, Heise L, Watts CH, Garcia-Moreno C (2008). Intimate partner violence and women’s physical and mental health in the WHO multi-country study on women’s health and domestic violence: an observational study. Lancet.

[8] Islam MJ, Baird K, Mazerolle P, Broidy L (2017). Exploring the influence of psychosocial factors on exclusive breastfeeding in Bangladesh. Archives of Women's Mental Health.

[9] Onigbogi MO, Odeyemi KA, Onigbogi OO (2015). Prevalence and factors associated with intimate partner violence among married women in an urban community in Lagos State, Nigeria. Afr J Reprod Health.

[10] Heise LL, Kotsadam A (2015). Cross-national and multilevel correlates of partner violence: an analysis of data from population-based surveys. Lancet Glob Health.

[11] Fleming PJ, McCleary-Sills J, Morton M, Levtov R, Heilman B, Barker G (2015). Risk factors for men’s lifetime perpetration of physical violence against intimate partners: results from the international men and gender equality survey (IMAGES) in eight countries. PLoS One.

[12] Rahman M, Nakamura K, Seino K, Kizuki M (2013). Does gender inequity increase the risk of intimate partner violence among women? Evidence from a National Bangladeshi Sample. PLoS One.

[13] Andrea L, Stephen E, Gilman G, Michele R, Decker K (2010). Witness of intimate partner violence in childhood and perpetration of intimate partner violence in adulthood. Epidemiology.

[14] White HR, Widom CS (2003). Intimate partner violence among abused and neglected children in young adulthood: the mediating effects of early aggression, antisocial personality, hostility and alcohol problems. Aggress Behav.

[15] Edleson JL (1985). Men who batter women: a critical review. J Fam Issues.

[16] Choice P, Lamke LK, Pittman JF (1992). Conflict resolution strategies and marital distress as mediating factors in the link between witnessing inter-parental violence and wife battering. Violence Vict.

[17] Charles L, Whitfield Robert F, Anda Shanta R, Dube V (2003). Violent childhood experiences and the risk of intimate partner violence in adults: assessment in a large health maintenance organization. J Interpers Violence.

[18] Uthman OA, Moradi T, Lawoko S (2011). Are individual and community acceptance and witnessing of intimate partner violence related to its occurrence? Multilevel structural equation model. PLoS One.

[19] Syeda KA, Sidra Z, Kashif S (2015). Is spousal violence being “vertically transmitted” through victims? Findings from the Pakistan Demographic and Health Survey 2012–13. PLoS One.

[20] Islam TM, Tareque MI, Tiedt AD, Hoque N (2014). The intergenerational transmission of intimate partner violence in Bangladesh. Glob Health Action.

[21] NIPORT, Mitra and Associates, and Macro International (2009). Bangladesh Demographic and Health Survey 2007.

[22] Straus MA (1979). Measuring intra-family conflict and violence: the Conflict Tactics (CT) Scales. J Marriage Fam.

[23] Fulu E1, Jewkes R, Roselli T, Garcia-Moreno C (2013). Prevalence of and factors associated with male perpetration of intimate partner violence: findings from the UN Multi-country Cross-sectional Study on Men and Violence in Asia and the Pacific. Lancet Glob Health.

[24] Kathryn M, Younta b, Eilidh H, Kristin E, Vander E, Hoang T. Men’s perpetration of intimate partner violence in Vietnam: gendered social learning and the challenges of masculinity. Men Masculinities 2015. Doi:10.1177/1097184X15572896.

[25] Kayoko Y, Tara MS, Krishna CP, Jimba M (2014). Acceptance of wife beating and its association with physical violence towards women in Nepal: a cross-sectional study using couple’s data. PLoS One.

[26] Edleson JL (1999). Children’s witnessing of adult domestic violence. J Interpers Violence.

[27] Cummings EM, El-Sheikh M, Kouros CD, Buckhalt JA (2009). Children and violence: the role of children’s regulation in the marital aggression-child adjustment link. Clin Child Fam Psychol Rev.

[28] Koenen KC, Moffitt TE, Caspi A, Taylor A, Purcell S (2003). Domestic violence is associated with environmental suppression of IQ in young children. Dev Psychopathol.

[29] Holtzworth-Munroe A, Meehan JC, Herron K, Rehman U, Stuart GL (2000). Testing the Holtzworth-Munroe and Stuart (1994) batterer typology. J Consult Clin Psychol.

[30] Dube SR, Anda RF, Felitti VJ, Edwards VJ, Williamson DF (2002). Exposure to abuse, neglect, and household dysfunction among adults who witnessed intimate partner violence as children: implications for health and social services. Violence Vict.

[31] Kitzmann KM, Gaylord NK, Holt AR, Kenny ED (2003). Child witnesses to domestic violence: a meta-analytic review. J Consult Clin Psychol.

[32] Rouse LP (1984). Models, self-esteem, and locus of control as factors contributing to spouse abuse. Victimology.

[33] Telch CF, Lindquist CU (1984). Violent versus nonviolent couples: a comparison of patterns. Psychotherapy Theory Res Pract Train.

